# A prospective cohort study to investigate cost-minimisation, of Traditional open, open fAst track recovery and laParoscopic fASt track multimodal management, for surgical patients with colon carcinomas (TAPAS study)

**DOI:** 10.1186/1471-2482-10-18

**Published:** 2010-06-14

**Authors:** Jurrian C Reurings, Willem R Spanjersberg, Henk JM Oostvogel, Erik Buskens, John Maring, Flip Kruijt, Camiel Rosman, Peter van Duivendijk, Cees HC Dejong, Cees JHM van Laarhoven

**Affiliations:** 1 Department of Surgery, Sint Elisabeth Hospital, Tilburg, the Netherlands; 2 Department of Surgery, Universitair Medisch Centrum Radboud., Nijmegen, the Netherlands; 3 Department of Epidemiology and Statistics, Universitair Medisch Centrum Utrecht, Utrecht, the Netherlands; 4 Department of Surgery, Tweesteden Hospital, Tilburg, the Netherlands; 5 Department of Surgery, Gelderse Vallei Ziekenhuis, Ede, the Netherlands; 6 Department of Surgery, Canisius Wilhelimina Ziekenhuis, Nijmegen, the Netherlands; 7 Department of Surgery, Academisch ziekenhuis Maastricht, P. Debyelaan 25, 6229 HX Maastricht, the Netherlands

## Abstract

**Background:**

The present developments in colon surgery are characterized by two innovations: the introduction of the laparoscopic operation technique and fast recovery programs such as the Enhanced Recovery After Surgery (ERAS) recovery program. The Tapas-study was conceived to determine which of the three treatment programs: open conventional surgery, open 'ERAS' surgery or laparoscopic 'ERAS' surgery for patients with colon carcinomas is most cost minimizing?

**Method/design:**

The Tapas-study is a three-arm multicenter prospective cohort study.

All patients with colon carcinoma, eligible for surgical treatment within the study period in four general teaching hospitals and one university hospital will be included. This design produces three cohorts: Conventional open surgery is the control exposure (cohort 1). Open surgery with ERAS recovery (cohort 2) and laparoscopic surgery with ERAS recovery (cohort 3) are the alternative exposures. Three separate time periods are used in order to prevent attrition bias.

Primary outcome parameters are the two main cost factors: direct medical costs (real cost price calculation) and the indirect non medical costs (friction method). Secondary outcome parameters are mortality, complications, surgical-oncological resection margins, hospital stay, readmission rates, time back to work/recovery, health status and quality of life.

Based on an estimated difference in direct medical costs (highest cost factor) of 38% between open and laparoscopic surgery (alfa = 0.01, beta = 0.05), a group size of 3×40 = 120 patients is calculated.

**Discussion:**

The Tapas-study is three-arm multicenter cohort study that will provide a cost evaluation of three treatment programs for patients with colon carcinoma, which may serve as a guideline for choice of treatment and investment strategies in hospitals.

**Trial registration:**

ISRCTN44649165.

## Background

### Usual care

The standard of care for colon carcinoma patients is changing. Traditionally operative treatment means an open resection (i.e. laparotomy) and 'conventional' patient-tailored recovery. In the last decade laparoscopic (assisted) colon surgery is introduced as a valuable alternative [1-5]. Over the last five years Enhanced Recovery After Surgery (ERAS) programs have been introduced and in many hospitals these alternatives gradually replace conventional care [6-11].

Equality of long term clinical (and oncological) effectiveness of laparoscopic surgery as well as ERAS recovery has been proven in literature [4,5,12-18]. To date however, strong evidence of cost minimization effects of these innovations is lacking.

Many hospitals are at a stage of investing large amounts in endoscopic operation theatres ('endosuites'). If laparoscopic surgery does not result in added cost effectiveness compared to open ERAS programs (or traditional open programs), future investments in endoscopic endosuites may be ill-advised. With this in mind a prospective cohort study is set up comparing traditional open, open-ERAS and laparoscopic-ERAS colon surgery.

### Health care problem

Costs of conventional open colon surgery is substantial. With approximately 9000 operations for colon carcinoma in the Netherlands annually, direct medical costs are estimated at 38.6 million euro [19].

Overall cost effectiveness studies on laparoscopic and open colon surgery have shown little differences [14,20]. This allows us to focus merely on direct medical costs accompanying laparoscopic surgery, showing an increase to 65.3 million euro for laparoscopic surgery (difference of 26.7 million euro per year). On top of these costs, hospital investments in laparoscopic operation theatres should be taken into account. Many Dutch hospitals are currently planning such investments. Reduction of time back to work (indirect non-medical costs) due to enhanced recovery from laparoscopy could compensate for these extra costs. However this cost minimization issue has never been clearly investigated.

Investment costs of ERAS recovery programs (mainly ward personnel costs and training facilities) have not been well established so far. Especially direct medical costs due to intensified activities of ward personnel should be considered. From a cost minimization point of view, reduction of hospital stay and enhanced rehabilitation to normal activity/work should equalize the increased personnel costs. This issue of presumed cost savings has not been raised so far nor evaluated.

In line with the two aforementioned cost minimization questions, a third question arises: which investment (in ERAS recovery programs, in laparoscopic operation theatres and -surgery or a combination of both) is most valuable for hospitals in near future.

## Methods/design

### Study objectives

The current study has the following two objectives:

To discern the most cost minimizing surgical peri-operative treatment program for colon cancer patients and to indicate justification or rejection of investments in these hospitals.

To supplement the lack of knowledge in literature on cost minimizing differences between the three treatment programs and to provide recommendations on further development in the operative treatment of colon carcinomas from an economic point of view.

### Study design

The Tapas-study is a multicenter prospective cohort study. Randomised Controlled Trials are considered to give the highest level of evidence. Since randomization produces the best comparable groups, which is methodological the best manner to compare the intervention group with the conventional/standard group. However, cohort studies can easily separate complex multidisciplinary treatment programs such as ERAS into time and place, preventing an important bias: the attrition bias. An attrition bias occurs when elements of the control and alternative intervention mix and hence influences outcome. This explains why studies with ERAS are often correctly designed as cohort studies and not as randomized controlled trials [21,22].

Conventional open surgery is the control exposure (cohort 1). Open surgery with ERAS recovery (cohort 2) and laparoscopic surgery with ERAS recovery (cohort 3) are the alternative exposures. Three separate consecutive time periods are used in order to prevent attrition bias. Before each new cohort starts a quality control assessment will be carried out by the expert project advisors from the research group on ERAS recovery and laparoscopic surgery.

### Primary and secondary endpoints

The primary endpoint of the Tapas-study are costs, both direct medical en indirect non medical costs. Direct medical costs are defined as the total costs of medical resources used for diagnostics, therapy, revalidation and peri-operative care. Indirect non medical costs are defined as the total costs due to loss of productivity measured by the time back to work.

Secondary endpoints are total postoperative hospital stay in days, including hospital stay ofpatients who are readmitted within 30 days after surgery and quality of life and health status after 6 weeks, 3 months and one year after surgery. Quality of life and health status will be measured by two validated questionnaires; Short Form 36 (SF-36) and the WHOQOL-100. Further secondary endpoints are complications and mortality within 30 days after surgery, readmission rates, time back to work and differences in surgical-oncological radicality between open and laparoscopic surgery.

### Participating centers

Five Dutch hospitals including one university medical centre en four general teaching hospitals, comparable by size and expertise, will enroll patients.

### Study population

The study population consists of all patients with colon carcinoma, eligible for surgical treatment within the study period January 2007 to July 2010.

Inclusion criteria are: patients with a primary colon carcinoma, over 18 years of age, ASA I en II, who give informed consent.

Exclusion criteria are: pregnancy, ASA III-IV, previous abdominal surgery and metastasized illness.

### Ethics

This study is conducted in accordance with the principles of the Declaration of Helsinki and 'good clinical practice' guidelines. All collected data will be kept confidential at all times. The independent medical ethics committees of all participating hospitals have approved the study protocol. Prior to inclusion, written informed consent will be obtained from all patients.

The proposed cohort study carries no additional risks, as extra diagnostics or other than established treatment methods will not be used.

### Study outline

Inclusion and informed consent is obtained at the outpatient clinic. The patient is presented the appropriate treatment modality according to the current cohort, after which informed consent for that specific treatment is obtained.

Patients assigned to cohort 2 and 3 with ERAS recovery will receive additional information about the ERAS program by an ERAS nurse and an anesthesiologist prior to hospital admittance. All patients in cohort 2 and 3 will be admitted to a ward, were nurses and medical staff are trained in the ERAS program, and the ERAS protocol used is checked by the project experts.

Patients included into the third cohort will be operated by an experienced laparoscopic surgeon. The requirement for surgeons to perform laparoscopic colon surgery is 20 completed procedures.

To accomplish standardization and quality of care of the participating hospitals, prior to the start of the study every clinic is visited and agreements will be made on peri-operative care and the surgical procedures. For cohort 1 a protocol which defines traditional/conventional care will be uses by every hospital. Before cohort 2, an ERAS-expert panel visits the hospitals to check and agree upon the ERAS elements used in the clinic. A minimum number of 8 ERAS elements or more will be considered as ERAS peri-operative care. Before cohort 3, a quality control assessment will be performed in every hospital by experts on laparoscopic colon surgery, also accomplishing standardization of the laparoscopic methods used.

### Discharge criteria

Whether the discharge criteria are met will be assessed and scored on every postoperative day. Discharge criteria include adequate pain control with oral analgesics, no nausea and ability to take solid foods, intestinal passage of flatus and/or stool, mobilization and self support as comparable to the preoperative level, agreement of the patient.

### Statistical analysis

#### Intention to Treat

The analysis will be performed in accordance with the intention to treat principle.

#### Sample size calculation

We used an extensive table to calculate all direct medical costs including overhead and consultant costs for open and laparoscopic colon surgery for an average Dutch hospital. The main cost differences were due to the difference in operating time, use of (disposable) laparoscopic instruments and hospital stay. Based on our costs calculation, for each patient the difference in direct medical costs between laparoscopic colectomy en open colectomy is 2967 euro (38%). Considering approximately 9000 colon carcinoma operations performed annually in the Netherlands http://www.prismant.nl, the hypothetical direct costs of all 'open' colectomies performed are 38.7 million euro. The hypothetical direct costs of laparoscopically colectomies for the Netherlands are 65.3 million euro. Performing all colectomies in the Netherlands by a laparoscopic technique would result in 26.7 (= 38%) euro extra direct costs compared to performing all colectomies by the open technique.

Power calculation of the study

p1 (direct medical cost of laparoscopic colon surgery in the Netherlands) = 100 %

p2 (direct medical cost of open colon surgery in the Netherlands) = 62%

alfa = 0.01, beta = 0.05, f = 17.8

Based on differences of approximately 2000 euro found in literature form other countries, the number needed in each group would be n = 43. Therefore a more conservative trial size of 3 × 40 = 120 patients is taken into account.

#### Economic evaluation

In the cost minimization study direct medical costs and the indirect non medical costs are estimated. For the direct medical costs the method of real cost price calculation will be used. This will include the additional costs of laparoscopy, of ERAS care, as well as the differences due to complications and readmission. For indirect non medical costs the friction cost method will be used [23]. This also allows us to limit the study period to one year post operatively. On the direct non medical costs and indirect medical costs assumptions of equality are made and these costs are not accounted for in this study.

### Data collection and monitoring

Data are collected in a specially designed case-record form, both in the outpatient clinic as during hospital stay. Preoperatively, as well as 6 weeks, 12 weeks and one year postoperatively quality of life questionnaires (SF-36/GIQLI, Vaizey) are filled in by the patient.

The overall study period is 30 months: three cohort periods followed by one year of data analysis and synthesis. Because of the end points used (i.e. direct medical costs and indirect medical costs due to rehabilitation to normal work/activity), a longer follow up is not indicated. At one year all important differences in recovery and rehabilitation to normal life and work can be analyzed. Middle and long term outcomes measures (like oncological recurrences and late secondary complications like adhesions and incisional hernias) will not be included. Histopathological result of resections will be evaluated and compared.

There will be regular contact between the study coordinators and the participating centers, and all included data will be monitored by a research fellow.

## Discussion

Extensive literature exists on ERAS and laparoscopic colon surgery. Regarding the comparison between ERAS recovery and conventional care level 1a evidence exists, showing at least equality and possibly improvement of clinical outcome [18]. Four level 1a reports exist on open versus laparoscopic colon surgery[2,3,12,17]: generally short term results of laparoscopic surgery are better, but no differences on middle and long term clinical outcome are found. Concern has existed on long term oncological outcome, but four level 1b studies (RCT's) have indicated equality in middle and long term (5 years) oncological outcome [4,13,15,16]. One level 1b study indicates that ERAS recovery is as effective in open and laparoscopic surgery, which theoretically biases and diminishes the superiority in short term results of laparoscopic compared to open surgery [6].

Abovementioned studies have convincingly shown that the results of ERAS recovery and/or laparoscopic surgery are at least comparable to those of the conventional surgical approach in colon carcinoma. Long term clinical results as well as surgical-oncological results are equal.

One RCT (level 1b) and three level 2b studies[14,20,24,25] on cost effectiveness of open versus laparoscopic surgery exist. Ignoring possible bias abovementioned, higher operation theatre expenses are leveled down by shorter hospital stays. Only one level 2b cost effectiveness study is available on open conventional recovery versus open ERAS recovery or the combined laparoscopic ERAS programs [26].

No ongoing studies focusing on cost minimization are reported so far. This study seeks to improve the lack of cost minimization data in literature, and may therefore provide important information on the justification of changing treatment policies and investments in hospital facilities for colon surgery.

## Competing interests

The authors declare that they have no competing interests.

## Authors' contributions

JCR drafted the manuscript. RS and CvL co-authored the writing of the manuscript. All other authors participated in the design of the study during several meetings and are local investigators at the participating centers. All authors edited the manuscript and read and approved the final manuscript.

## Pre-publication history

The pre-publication history for this paper can be accessed here:

http://www.biomedcentral.com/1471-2482/10/18/prepub
